# Highly thermal-stable ferromagnetism by a natural composite

**DOI:** 10.1038/ncomms13937

**Published:** 2017-01-18

**Authors:** Tianyu Ma, Junming Gou, Shanshan Hu, Xiaolian Liu, Chen Wu, Shuai Ren, Hui Zhao, Andong Xiao, Chengbao Jiang, Xiaobing Ren, Mi Yan

**Affiliations:** 1 School of Materials Science and Engineering, State Key Laboratory of Silicon Materials, Key Laboratory of Novel Materials for Information Technology of Zhejiang Province, Cyrus Tang Center for Sensor Materials and Applications, Zhejiang University, Hangzhou 310027, China; 2 Ferroic Physics Group, National Institute for Materials Science, 1-2-1 Sengen, Tsukuba 305-0047, Japan; 3 Multi-disciplinary Materials Research Center, Frontier Institute of Science and Technology, Xi'an Jiaotong University, Xi'an 710049, China; 4 School of Materials Science and Engineering, Beihang University, Beijing 100191, China

## Abstract

All ferromagnetic materials show deterioration of magnetism-related properties such as magnetization and magnetostriction with increasing temperature, as the result of gradual loss of magnetic order with approaching Curie temperature *T*_C_. However, technologically, it is highly desired to find a magnetic material that can resist such magnetism deterioration and maintain stable magnetism up to its *T*_C_, but this seems against the conventional wisdom about ferromagnetism. Here we show that a Fe–Ga alloy exhibits highly thermal-stable magnetization up to the vicinity of its *T*_C_, 880 K. Also, the magnetostriction shows nearly no deterioration over a very wide temperature range. Such unusual behaviour stems from dual-magnetic-phase nature of this alloy, in which a gradual structural-magnetic transformation occurs between two magnetic phases so that the magnetism deterioration is compensated by the growth of the ferromagnetic phase with larger magnetization. Our finding may help to develop highly thermal-stable ferromagnetic and magnetostrictive materials.

## Abstract (web summary)

Magnetism deterioration is usually expected in all ferromagnetic materials with increasing temperature. Here, Ma *et al*. report a Fe-Ga alloy with highly thermal-stable magnetization up to 880 K and with nearly no deterioration over a wide temperature range in magnetostriction.

Technologically, magnetic materials that can resist magnetism deterioration when operating at elevated temperatures are highly desirable in applications of aircraft, space and defence systems^1^,^2^,^3^,^4^,^5^. Magnetization of all single-phase ferromagnets, however, decreases when approaching *T*_C_ as the thermal energy disrupts the magnetic ordering. The relative magnetization *m*, *M*/*M*(0), decreases with relative temperature *T*/*T*_C_, following the Brillouin function^6^,^7^. Although some ferrimagnets that contain two ferromagnetic sublattices can even exhibit positive temperature coefficient of magnetization over a certain temperature range, their net magnetization also inevitably deteriorates when approaching *T*_C_. Consequently, high *T*_C_ is usually required to ensure sufficiently high operating temperature. For instance, *T*_C_ of the advanced soft and hard magnets is 1,203 K for Fe–Co–V alloy^2^ and 1,093 K for 2:17-type Sm–Co compound^3^, respectively, which maintain the magnetic performance when operating at temperatures above 773 K. For a given magnetic material, the usual intrinsic approach to achieve thermally stable magnetization is to enhance *T*_C_ through alloying. It may, however, deteriorate other related magnetic properties, for example, the magnetostriction that is a fundamental property of ferromagnets referring to the macrostrain induced by an external magnetic field. For instance, alloying with Co enhances *T*_C_ of the giant magnetostrictive materials Tb–Dy–Fe (Terfenol-D) by ∼50 K at the expense of decreasing room temperature magnetostrictive property^8^,^9^,^10^. Extrinsically, positive temperature coefficient of magnetic anisotropy can be achieved in a hybrid magnetic/polymer structure, for which the anisotropic thermal expansion of polymer results in enhanced magnetic anisotropy at elevated temperatures^11^. Nevertheless, temperature-independent magnetization has never been achieved in any single-phase ferromagnetic material since it is against the basic law of magnetism. In this work, we report an unusual finding that nearly temperature-independent magnetization and thermally stable magnetostriction exist in a natural ferromagnetic composite, the Fe–Ga alloy.

Since the discovery in 2000, mechanically strong and malleable Fe_100−*x*_Ga_*x*_ alloys (known as Galfenol) have attracted intensive attention due to the combination of large magnetostriction, low switching field and high *T*_C_, which are promising for applications in sensors, actuators and transducers^12^,^13^,^14^,^15^,^16^,^17^,^18^,^19^,^20^,^21^,^22^,^23^,^24^,^25^,^26^,^27^,^28^. Previous investigations^18^,^20^,^21^,^22^,^23^,^24^ have shown that both magnetization and structure of the Fe_100−*x*_Ga_x_ alloys are very sensitive to heat treatments. Various structures including disordered body-centred cubic (bcc) A2, ordered bcc B2 and DO_3_, ordered face-centred cubic (fcc) L1_2_, and hexagonal DO_19_ (hcp) can be obtained when *x* is ∼27 (∼26–29). Among these phases, L1_2_ possesses the largest magnetization. Formation of the L1_2_ phase is usually based on a kinetically slow diffusion transformation from bcc precursors^26^,^27^,^28^, for instance, long-term aging (3 days annealing at 773 K followed by 1 month at 623 K (ref. 21)) or slow cooling (at 0.1 K min^−1^ (ref. 25)) after solution treating in high-temperature bcc phase regime (the obtained bcc phases are metastable) have been performed. Controlling the aging time gives rise to the formation of a composite, in which the remanent bcc phase coexists with the transformed L1_2_ phase.

In the following, we shall show that an aged Fe_73_Ga_27_ alloy, containing both L1_2_ phase and remanent bcc phases, exhibits highly thermal-stable magnetization up to its *T*_C_ (∼880 K) and stable magnetostriction over a wide temperature range. Such unusual behaviours stem from simultaneous magnetic/structural transformations from bcc into L1_2_, which offsets the thermally induced magnetization reduction. The highly thermal-stable magnetization and magnetostriction may lead to numerous high-temperature applications.

## Results

### Magnetization and magnetostriction temperature dependence

**Figure 1:**
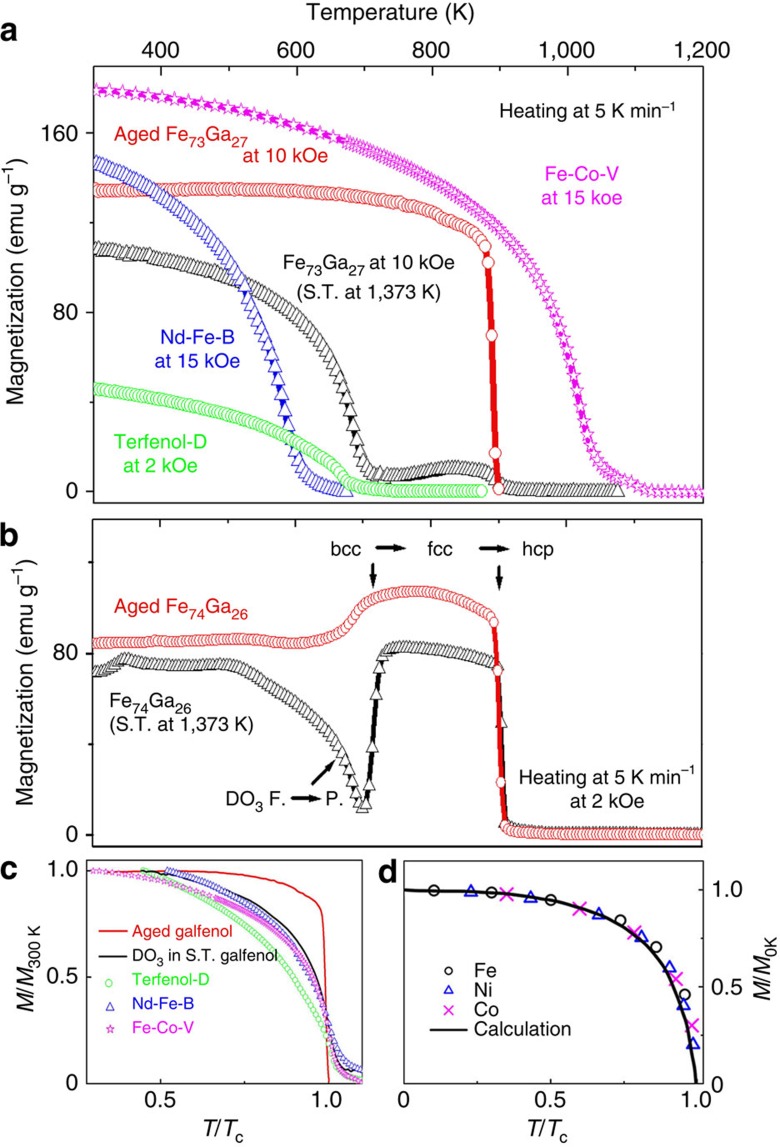
Magnetization temperature dependence. (**a**) *M*−*T* curves for the solution-treated and the aged Fe_73_Ga_27_, Terfenol-D (Tb_0.3_Dy_0.7_Fe_2_), Nd–Fe–B and Fe–Co–V commercial magnets. (**b**) *M*–*T* curves for another proof-of-principal reference, Fe_74_Ga_26_ subjected to the same heat treatments with Fe_73_Ga_27_. The abbreviated S.T. denotes solution-treated, while F. means ferromagnetic and P. means paramagnetic. (**c**) Relative magnetization as a function of relative temperature for the *M*−*T* curves in **a**. (**d**) Normalized *M*−*T* curves for Fe, Co and Ni metals (scattered symbols) and calculation using the Brillouin function with *J*=1/2 (black curve; redrawn from ref. 7).

Figure 1a shows the *M*−*T* curves for Fe_73_Ga_27_ alloy subjected to different heat treatments (sample *A* was solution-treated at 1,373 K, bearing a bcc average structure and sample *B* was further aged for 12 h at 803 K after solution treatment, containing both bcc and fcc phases), the well-known Terfenol-D giant magnetostrictive materials (ferrimagnetic, *T*_C_∼663 K (ref. 29)), Nd–Fe–B (the strongest permanent magnet, *T*_C_∼585 K), and Fe–Co–V (*T*_C_∼1,014 K) commercial magnets. The solution-treated sample (black curve) suffers gradual magnetization deterioration upon heating to ∼700 K, which is similar to normal Curie transition. A slight enhancement from 700 to 880 K is observed, followed by rapid reduction in magnetization. The aged sample (red curve), however, exhibits unusual weak temperature dependence of magnetization up to 880 K, above which the net magnetization drops rapidly to nearly zero. The thermal cycling *M*−*T* curves and isothermal *M*−*H* curves for the aged sample were also measured. Over the temperature range from 310 to 850 K, the magnetizations in both low and high magnetic fields (2 and 10 kOe, respectively) are reproducible and stable up to five thermal cycles (Supplementary Fig. 1). Especially, there is no obvious tendency of magnetization deterioration observed for the 2 kOe case. The low-field *M*−*H* curves (Supplementary Fig. 2) are nearly overlapped with each other for all the measured temperatures ranged from 298 to 773 K. Such highly thermal-stable magnetization has not been observed in any single-phase ferromagnet. Also in Fig. 1a, ferromagnetic Nd–Fe–B and Fe–Co–V, and ferrimagnetic Terfenol-D lose magnetization gradually upon heating, despite that they have either higher or lower *T*_C_ than the aged Fe_73_Ga_27_. The highly thermal-stable magnetization is reproduced in another proof-of-principal reference, Fe_74_Ga_26_ alloy subjected to the same heat treatments (Fig. 1b). The normalized *M*−*T* curves in Fig. 1c directly show the differences among them. In comparison with the reference magnets, where *m* decreases gradually from 1 to 0 when approaching to their *T*_C_, *m* for the aged sample remains at relatively larger values, exhibiting totally different temperature dependence. According to the Weiss theory, *m* as a function of relative temperature for a single-phase ferromagnet can be fitted well by the Brillouin function (the solid line in Fig. 1d), which agrees well with experiments of Fe, Ni and Co metals^6^,^7^. It is noted that *T*_C_ for the aged Fe_73_Ga_27_ is a critical temperature, at which the absolute value of d*M*/d*T* reaches a maximum.

**Figure 2:**
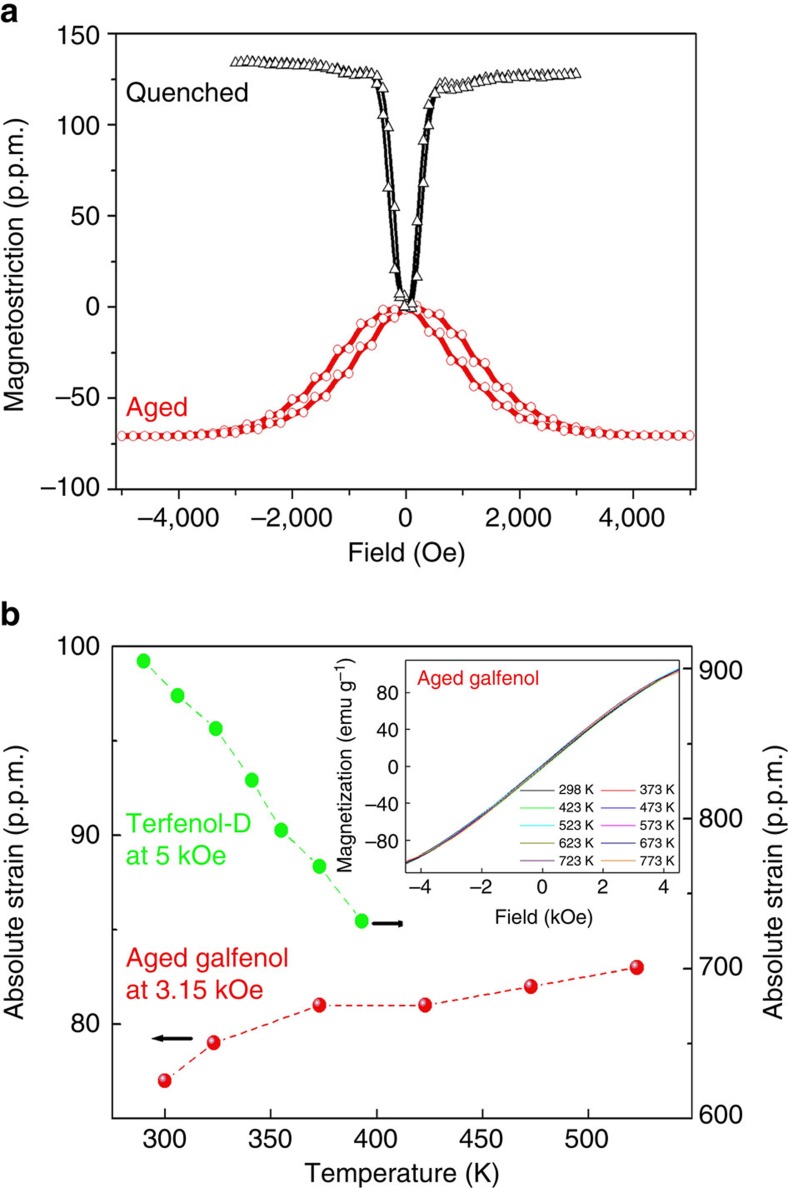
Magnetostriction and its thermal stability. (**a**) Room temperature (298 K) magnetostrictions for the solution-treated and aged Fe_73_Ga_27_. (**b**) Magnetostriction temperature dependence for the aged Galfenol (red symbols) and the comparison with Terfeonl-D (green symbols). Inset in **b** illustrates the isothermal magnetization loops for the aged Galfenol at low magnetic fields.

The aged Fe_73_Ga_27_ also exhibits thermally stable magnetostriction over a wide temperature range. At room temperature, the saturation magnetostriction is positive 130 p.p.m. for the as-solution-treated state, but is negative 70 p.p.m. for the as-aged state (Fig. 2a). In addition, the aged sample has stronger magnetocrystalline anisotropy than the solution-treated one, as the saturation field and magnetostriction hysteresis are larger than those for the latter. According to earlier studies^14^,^22^,^23^,^30^,^31^, bcc (A2, B2, and DO_3_)-structured Fe–Ga alloys have positive magnetostriction, but the fcc (L1_2_)-structured Fe–Ga alloys have negative magnetostriction. The positive magnetostriction is consistent with these studies. The negative magnetostriction for the aged samples is an offset result between the bcc and fcc phases because the fcc phase becomes the majority, as revealed by the X-ray diffraction profiles (later shown in Fig. 4). The enlarged magnetostriction hysteresis is because that the formation of L1_2_ phase will enlarge the magnetocrystalline anisotropy^31^. In comparison with Terfenol-D, for which the magnetostriction decreases with increasing temperature, the aged Galfenol, however, shows very thermally stable magnetostriction (Fig. 2b). The absolute magnetostriction measured by standard strain gauge method shows a positive temperature coefficient upon *in situ* heating up to 523 K (the upper temperature limit of the strain gauge). As the linear magnetostriction in ferromagnets is a consequence of motion of non-180° domain walls and rotation of magnetization under magnetic fields, the magnetostriction thermal stability of the Fe_73_Ga_27_ composite at temperatures above 523 K can then be reflected by the isothermal magnetizations. The magnetostriction nearly saturates at 4.5 kOe (Fig. 2a), below this field, the magnetizations (the inset of Fig. 2b) are nearly unchanged as the sample is heated up to 773 K. The stable magnetization implies stable magnetostriction over a wide temperature range. In addition, the magnetostriction does not deteriorate after thermal cycling, as shown in Supplementary Fig. 3. After four thermal cycles (one cycle means that heating from 310 to 850 K and cooling back to 310 K), the absolute magnetostriction (at room temperature) is slightly larger than the initial one.

Consequently, the following question arises, what is the mechanism underlying the unusual highly thermal-stable magnetization and magnetostriction observed in the aged Galfenol? To answer this question, we have investigated the structural evolutions upon *in situ* heating as follows.

### Structural transitions

**Figure 3:**
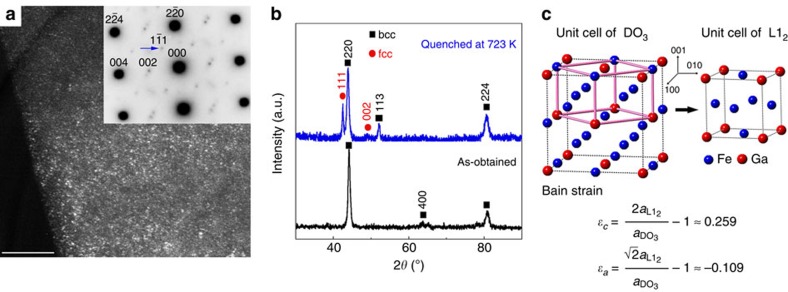
Structural characterization of the solution-treated Fe_73_Ga_27_. (**a**) Dark-field image taken using the 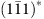 superlattice reflection, inset is the corresponding SAED pattern with [110] zone axis. (**b**) X-ray diffraction patterns for the as-obtained sample (black) and the one quenched after annealing for 0.5 h at 723 K (blue). The reflections are indexed by taking DO_3_ (space group 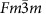) as a reference (abbreviated as bcc), the additional reflections are for L1_2_ (abbreviated as fcc). (**c**) Bain relation between DO_3_ and L1_2_ structures. Scale bar, 50 nm (**a**).

X-ray diffraction profiles in Fig. 3 reveal an average bcc structure at the solution-treated state. The calculated lattice constant is 0.2930, nm for the disordered A2 (ordered B2) structure or 0.5860, nm for the ordered DO_3_ structure (Fig. 3b). According to previous work^19^,^20^,^32^, the solution-treated Fe–Ga alloys with *x*∼25–29.9 contain mixed phases of A2+B2+DO_3_, all of them have smaller magnetization than L1_2_. For easy understanding, we take DO_3_ to index the reflections of bcc phases. {111} superlattice reflections in the selected area electron diffraction (SAED) pattern (inset of Fig. 3a) demonstrate the existence of ordered DO_3_ phase, which is randomly distributed as nanodomains (dark-field image, Fig. 3a). The intensity of {111} reflections, however, is weaker than the {200} ones that contributed from both B2 and DO_3_ phases, indicating that B2 phase coexists. In addition, the fundamental reflections of A2, B2 and DO_3_ phases could be overlapped with each other, hence the existence of A2 cannot also be excluded. The *M*−*T* curves (black) in Fig. 1a clearly show that the first magnetization drop at ∼700 K corresponds to the Curie transition of DO_3_ phase^27^, where the net magnetization reduces by over 80 % when compared with that at 300 K, indicating that the DO_3_ phase has a relatively large volume fraction. The following changes are consistent with the equilibrium phase diagram^19^, which are the bcc→L1_2_ and ferromagnetic L1_2_→paramagnetic DO_19_ transformations, respectively^27^,^28^,^33^. Evidence for the bcc→L1_2_ transformation is presented by the blue curve in the X-ray diffraction data in Fig. 3b, which was taken from a piece quenched from 723 K (it is heated to 723 K at 5 K min^−1^ before quenching). Based on the Bain relation (Fig. 3c), the transformation from DO_3_ into L1_2_ is via (i) large lattice strain (*ɛ*_[001]DO3_∼0.259 and *ɛ*_[010]DO3_∼−0.109) and (ii) atomic position exchanges (Fe or Ga change sites). The complete structural transformation then requires long-term diffusion to promote atom migrations.

**Figure 4:**
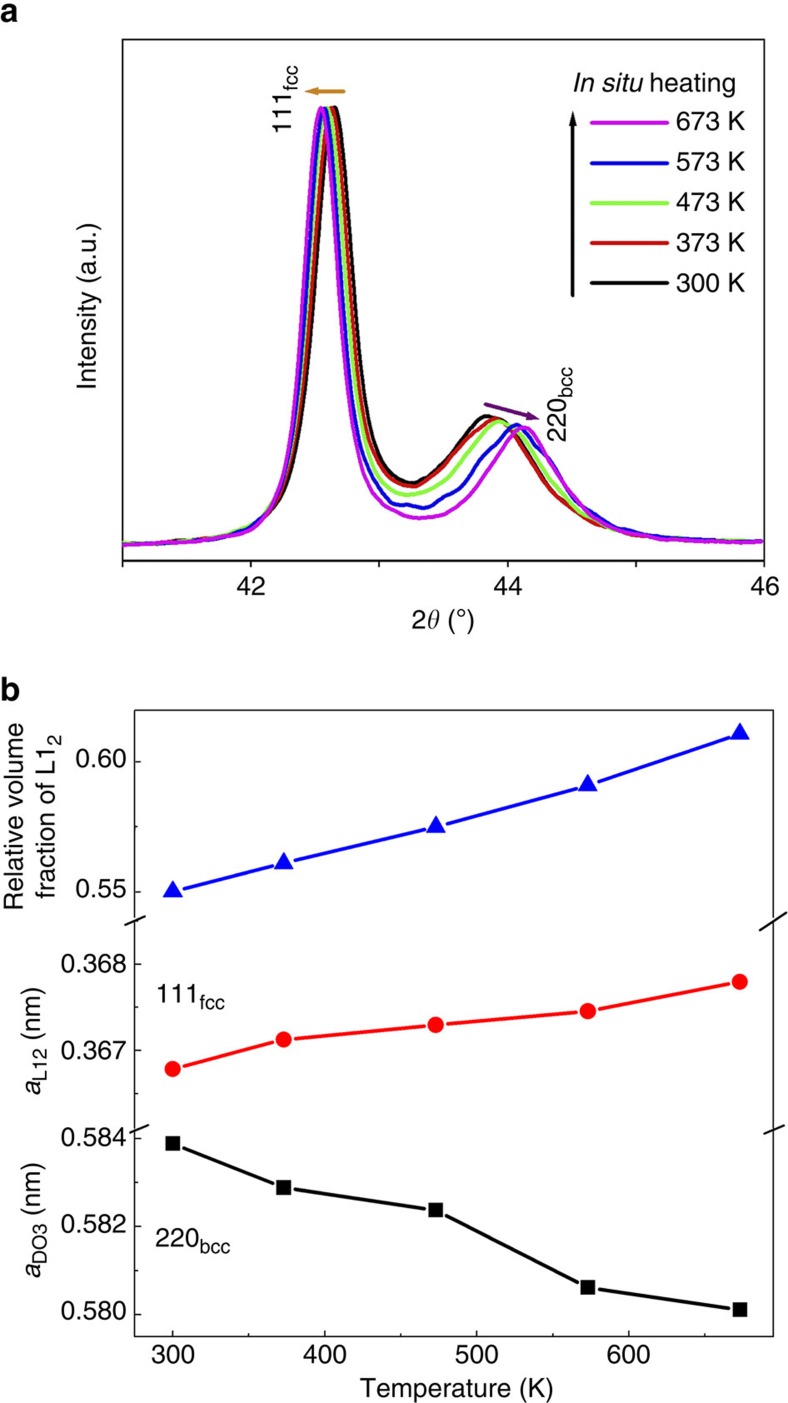
Average structure evolution of the aged Fe_73_Ga_27_. (**a**) *In situ* heating step-scanned X-ray diffraction profiles with 2*θ* from 41 to 46°, showing the {111} reflection for L1_2_ phase and the {220} reflection for DO_3_ phase, respectively. Arrows indicate the shifts of Bragg angle upon heating. (**b**) Temperature dependences of relative volume fraction of L1_2_ phase and lattice parameters for the two phases.

X-ray diffraction profiles illustrated in Fig. 4a reveal that the Fe–Ga composite is formed after aging, exhibiting much larger magnetization than the solution-treated state (Fig. 1a). The strong {111}_fcc_ reflections and weakened {220}_bcc_ reflections indicate that L1_2_ phase accounts for the majority and bcc phases are the minorities. The lattice constant *a*_L12_ is 0.3668, nm at 300 K. Upon *in situ* heating, the remanent bcc phase continues to transform into the L1_2_ phase. The relative volume fraction of the L1_2_ phase, which is estimated from the {111}_fcc_ reflection and {220}_bcc_ reflection fitted by Gauss function, increases gradually from 56.1 % at 300 K to 61.2 % at 673 K (Fig. 4b). Although such estimation is taken from the X-ray diffraction profiles of the sample surface that may not totally reflect the bulk effect, it indeed demonstrates that the remaining bcc phase will continually transform into fcc phase upon heating. In addition, the {111}_fcc_ reflection shifts towards lower Bragg angles, meanwhile the {220}_bcc_ one shifts to higher Bragg angles. Despite the thermal expansion effect, lattice contraction of the bcc phase (gradual decrease of *a*_DO3_, Fig. 4b) also demonstrates the occurrence of diffusion-type structural transformation with composition change. Since Ga has a larger atomic radius than Fe (140 pm for Ga and 127 pm for Fe), it can be inferred that the bcc→L1_2_ transformation results in the decrease of Ga concentration in the bcc phase. Such structural transformation then offsets the thermally reduced magnetization of the untransformed components. As the volume fraction of L1_2_ phase increases, the net magnetization at 2 kOe becomes even larger than the low temperature ranges, as shown in Supplementary Fig. 1. It should be addressed that the magnetic field strength has a strong influence on the bcc→L1_2_ diffusion-type transformation. For the *M*−*T* curve measured under 2 kOe (Fig. 1b), the net magnetization over the temperature range from 700 to 880 K is even higher than that of 300 K for the aged sample. However, under a stronger magnetic field of 10 kOe, only slight magnetization increment over this temperature range is observed. It indicates that the strong magnetic field restricts such diffusion-type magnetic/structural transformation, stabilizing the bcc phase.

**Figure 5:**
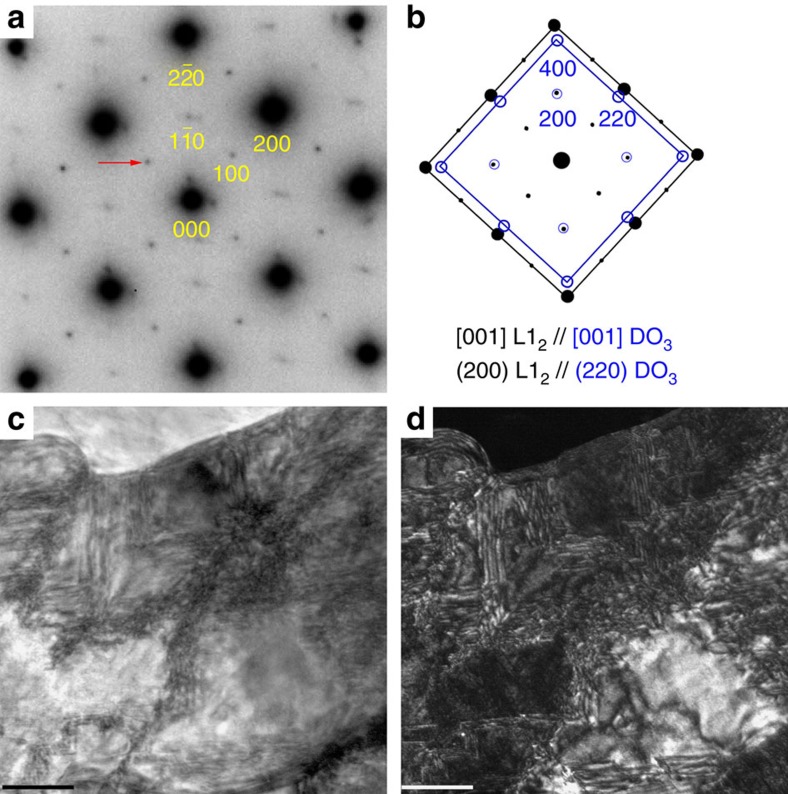
Room temperature microstructure of the aged Fe_73_Ga_27_. (**a**) SAED pattern with [001]_fcc_ zone axis, showing the matrix L1_2_ phase and the secondary bcc phase. (**b**) Orientation relationship between L1_2_ (black) and DO_3_ (blue) phases. (**c**) Bright-field image taken with [001]_fcc_ incidence. (**d**) Dark-field image taken using the 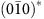
_fcc_ superlattice reflection, indicated by red arrow in **a**. Scale bars, 200 nm (**c**,**d**).

**Figure 6:**
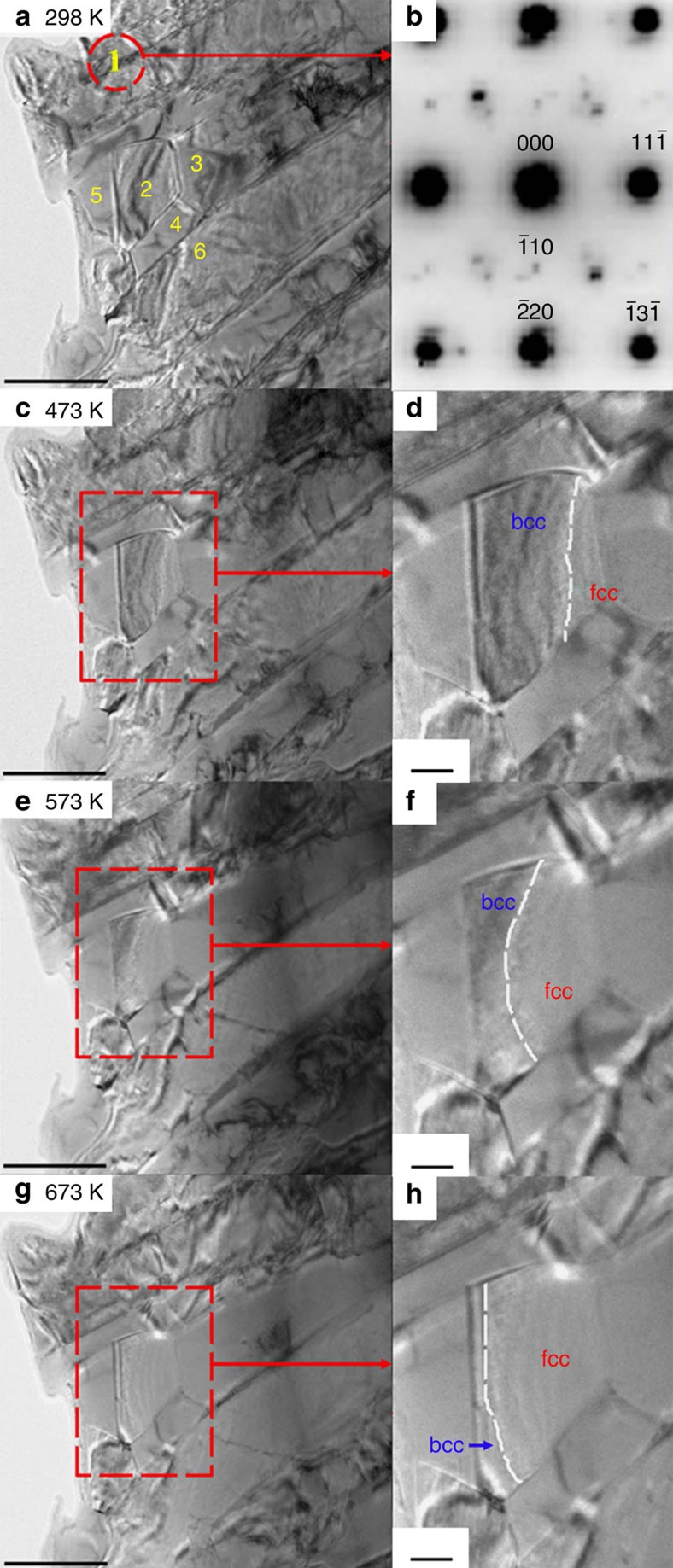
Microstructure evolution for the aged Fe_73_Ga_27_. In situ TEM characterizations at (**a**,**b**) 298, (**c**,**d**) 473, (**e**,**f**) 573 and (**g**,**h**) 673 K, respectively. All bright-field images are taken by tilting grain 1 (dashed red circle region) with the [112]_fcc_ zone axis, the SAED pattern taken at 298 K is shown in (**b**). (**d**, **f**, **h**) are enlarged views of the red rectangles in (**c**,**e**,**g**), respectively, where the interface between transformed (indicated by fcc) and untransformed (indicated by bcc) regions in grain 2 is indicated by dashed white line, showing that the volume fraction of fcc phase increases upon heating. Scale bars, 500 nm (**a**,**c**,**e**,**g**); 100 nm (**d**,**f**,**h**).

In the SAED pattern (Fig. 5a), superlattice reflections appear at both {100}* and {110}* positions, verifying the L1_2_ ordered structure. The remanent bcc phase coexists even inside an individual grain. Weak additional spots are visible around the four fundamental {220}_L12_ ones, diffuse scatterings also appear for the four fundamental {200}_L12_ reflections and the four {110}_L12_ superlattice ones. In addition, the {110}_L12_ superlattice spots are not as sharp as the {100}_L12_ ones. As shown by the crystallographic relationship (Fig. 5b), the [001] axes of both L1_2_ and the remanent bcc phases are parallel to each other, the (200)_L12_ plane is parallel to the (220)_DO3_. Consequently, the satellite spots are the {400} reflections for DO_3_ phase, and the diffusing scatterings are ascribed to the overlap of {200}_L12_ reflections by {220}_DO3_ and the overlap of {110}_L12_ by {200}_DO3_. The typical grain size (Fig. 5c; Fig. 6a) is from several hundreds of nanometres to several microns. The dark-field image taken using one {100}_L12_ superlattice reflection (which arises from the L1_2_ phase only) shows the coexistence of bcc and fcc phases. A large amount of tiny sheet-like L1_2_ nanodomains are revealed in the dark-field image (Fig. 5d). The *in situ* heating transmission electron microscopy (TEM) characterizations in Fig. 6a–d show directly structural transformation in a local region. The bright-field image highlights several grains numbered with 1–6. The enlarged views clearly show shift of the interface between transformed and untransformed regions. At 473 K, the interface is close to the grain boundary G2/G3, but gradually shifts near the boundary G2/G5 when heating to 673 K. The neighbouring grain 4 also exhibits a similar transformation, inside which the interface shifts towards the bottom-left corner when heating to 673 K. This gradual structural transformation (from the one with lower *M*_S_ into the one with larger *M*_S_) is responsible for the nearly balanced net magnetization over this temperature range. The slight magnetization reduction in Fig. 1 at temperatures close to 880 K is due to the thermal-related ordering loss for the fcc phase, whose *T*_C_ is predicted as high as *α*-Fe, ∼1,042 K (refs 2, 14). However, the ferromagnetic fcc→paramagnetic hcp transformation disrupts completely magnetic ordering before its *T*_C_.

## Discussion

To understand the thermally stable magnetization, the structural transformation between two conventional ferromagnetic phases (bcc→L1_2_) in the Fe_73_Ga_27_ alloy is essential. From the equilibrium Fe–Ga phase diagram^19^, the phase equilibria of this alloy is L1_2_. The solution-treated sample is in metastable state, where A2, B2 and DO_3_ phases coexist, as shown by the SAED pattern (inset of Fig. 3a). Further aging promotes the formation of L1_2_ phase. According to the model proposed by Khachaturyan *et al*.^26^, the transformation from DO_3_ into L1_2_ is via a face-centered-tetragonal (fct) DO_22_ intermediate structure. A drastic shearing of the {110} plane is also required for this transformation. The large energy barrier (the formation energy is −165 meV per cell for DO_3_, ∼−150 meV per cell for DO_22_, and ∼−340 meV per cell for L1_2_, respectively^34^) as well as the different atomic occupations of Fe and Ga between DO_3_ and L1_2_ structures (Fig. 3c) determine that DO_3_→L1_2_ is a diffusion-type transformation with very slow kinetics. Consequently, the whole transformation includes the nucleation of fct embryos within the bcc matrix, the change of the tetragonality towards the fcc structure and the growth of the new phase domains. Due to the slow transformation kinetics, the Fe_73_Ga_27_ alloy aged for 12 h at 803 K does not reach the phase equilibria state (Fig. 4). Upon heating, the remaining bcc phases will continually transform into the fcc phase with larger magnetization. This is why the aged sample has thermally stable magnetization as well as the stable magnetostriction. Furthermore, the remanent bcc phases still exist even after aging for 30 days at 803 K (ref. 31). *M*–*T* curve for the 30-day-aged sample also reveals thermally stable magnetization below 880 K (Supplementary Fig. 4), indicating that the bcc→L1_2_ transformation is incomplete. It also implies that the Fe_73_Ga_27_ alloy aged for 12 h at 803 K may have long operating life. Of course, one should not deny that as more thermal cycling is proceeded, the remanent bcc phases will no longer transform into L1_2_ phase, for instance, Ga content in the bcc phase could exceed the narrow composition range (Ga content is within 26–29 at.%) where the L1_2_ phase can be formed. Once the complete transformation is reached, it should not bear such thermally stable magnetization any more, that is, the magnetization decreases with temperature. Evidence may see from an early study^21^, in which L1_2_-structured Fe_73_Ga_27_ was obtained by heat treating for 3 days at 1,373 K and subsequent annealing for 3 days at 773 K and for 1 month at 623 K and its magnetization follows conventional degradation with temperature prior to the L1_2_→DO_19_ transformation.

The fast drop in magnetization around *T*_C_ is ascribed to the L1_2_→DO_19_ transformation, as observed in both the as-solution-treated and the aged samples (Fig. 1; Supplementary Fig. 4). In fact, this transformation follows well the Fe–Ga equilibrium phase diagram^19^. Such transformation has also been verified by an *in situ* heating neutron diffraction study^33^ and a quasi *in situ* X-ray diffraction study^27^. As reported in a similar alloy Fe_3_Ge (ref. 35), the numerous stacking fault debris and twining boundaries existed in the L1_2_ phase act as efficient nucleation site for DO_19_ phase by a shear-type mechanism. Consequently, the L1_2_→DO_19_ transformation is very fast, which is observed to be of the order of 300 s. The *in situ* neutron diffraction study by Golovin *et al*.^33^ shows that the L1_2_→DO_19_ transformation in the Fe_73_Ga_27_ alloy between two closed packed lattices takes place in very small temperature intervals (Δ*T*≈10 K), demonstrating its fast transformation kinetics, and they also observed a similar sudden decrease of magnetization. Consequently, the Curie transition of the present Fe_73_Ga_27_ composite involves a rapid L1_2_→DO_19_ structural transformation by nature.

The above unconventional thermal behaviour of magnetization is shown to stem from a gradual structural transformation from one phase with smaller magnetization into another with larger magnetization, which compensate for the natural magnetization degradation with temperature; this mechanism has not been reported before and its result may have profound technological implications. First, it provides new insights into the magnetism temperature dependence. According to the basic law of ferromagnetism, magnetization of a single-phase ferromagnet deteriorates when approaching *T*_C_. Our results clearly show that the solid-state structural transformation in the magnetic composite can resist the magnetization deterioration. It is also different from the ferrimagnets, for which moments of two ferromagnetic sublattices are antiparallel to each other at the atomic scale. Despite that the offset between two sublattices can result in positive temperature dependence of magnetization, each sublattice follows the Brillouin function and loses magnetic ordering above *T*_C_ finally^6^,^7^. It should be noted that although the individual phase follows a Billouin-function-type magnetization degradation with temperature, the unique feature of the present Fe–Ga composite, that is, the coexistence of two ferromagnetic components and the gradual structural transformation upon heating, enables the surprising phenomenon of highly stable magnetization of the composite. Consequently, the mechanism behind the thermal-stable magnetization is not a simple rule of mixture of two stable phases, but a mixture of two inter-transforming phases.

Second, the highly thermal-stable magnetostriction may facility novel applications at elevated temperatures. Magnetostriction is an important function of ferromagnets, referring to the strain generated by external magnetic field. *T*_C_ is the upper temperature limit of the magnetostriction for all single-phase ferromagnets. As shown in Fig. 2, the magnetostriction deteriorates gradually for Terfenol-D that possesses the largest room temperature magnetostriction among all single-phase ferromagnets. However, in the aged Galfenol, the magnetostriction deterioration is suppressed by the underlying structural transformation. Such highly thermal-stable magnetostriction then makes the Galfenol composite as promising candidate in sensors, actuators and transducers with high operating temperature. In addition, Galfenol is known for its good mechanical properties and low coercivity^11^, the high thermal stability of the present composite may also make it as a potential candidate for magnetic bearings or magnetic rotors used in high-temperature space power or engine systems.

In summary, the highly thermal-stable magnetization over a very wide temperature range has been discovered in a natural composite, the proof-of-principal reference Fe–Ga alloy. The metastable nature of the bcc component (that has lower magnetic ordering, smaller magnetization) ensures simultaneous magnetic/structural transformations into the fcc L1_2_ phase (that has higher magnetic ordering, larger magnetization) to offset the net magnetization deterioration. It facilities excellent thermal stability of the magnetostrictive performance. Consequently, the present work offers a novel approach to design thermally stable ferromagnetic and magnetostrictive materials by natural composites which may lead to potential applications.

## Methods

### Material processing and sample preparation

Ingots with nominal compositions Fe_73_Ga_27_ and Fe_74_Ga_26_ were prepared by arc-melting Fe and Ga metals with purity of 99.99% in argon atmosphere. Sheet-like samples were prepared by subsequent hot-rolling at 1,273 K. Pieces with thickness of 2 mm were sealed in quartz tubes filled with high-purity argon. Solution treatment was performed for 3 h at 1,373 K, followed by quenching into oil. After solution treating, another specimen was subjected to further aging for 12 h at 803 K, and final quench into oil. Specimen surfaces for structural and magnetic characterizations were carefully treated after removing damaged surfaces by colloidal amorphous silica polishing^36^ and final Nital solution etching. To avoid the possible texture effect for the hot-rolled sample, a reference specimen was also prepared by arc-melting, followed by the same solution treating and aging processes, which also produce a ferromagnetic composite containing both bcc and fcc phases (Supplementary Figure 5 illustrates the X-ray diffraction patterns for this specimen). The magnetic measurements in Supplementary Fig. 1 are based on this specimen.

### Characterization methods

The average structure was characterized by X-ray diffraction on a Rigaku diffractometer with Cu *K*α radiation. Continuous-scanned profiles with 2*θ* from 30 to 90° were obtained at 2° min^−1^. Step-scanned profiles with 2*θ* from 41 to 46° were obtained with each step of 0.01° staying for 8 s. Since {111} and {220} reflections have the strongest intensities for the L1_2_ and the DO_3_ structures, respectively, the relative volume fractional of the L1_2_ phase in the aged sample is estimated by fitting the X-ray diffraction profiles in Fig. 4a. The foils for TEM observation were prepared by twin-jet electropolishing at temperatures below 243 K, followed by ion-milling for 15 min (beam glancing angle=6°, beam voltage=2 keV and beam current=5 mA) to remove surface contaminations. TEM observation was performed using a JEOL JEM-2100F microscope. *In situ* heating TEM characterizations were conducted using a heating holder. It is noted that the structural transformation within a thin film and a bulk material could be different, but the gradual structural transformation in this alloy can be clearly revealed. Temperature dependence of magnetization was measured by a Lakeshore-7407 vibrating sample magnetometer to identify the magnetic transitions upon heating to 1,273 K. Isothermal magnetization hysteresis loops (Supplementary Fig. 2 and the inset of Fig. 2b) were obtained by *in situ* heating the samples to different temperatures. Each fresh sample size for magnetic measurements is 2 × 2 × 2 mm^3^. Magnetostriction was measured using strain gauge method. The sample size is 15 × 6 × 2 mm^3^, both measurement direction and magnetic field are along the length direction. To obtain the magnetostriction at elevated temperatures (the results in Fig. 2b), a strain gauge with upper temperature limit of 523 K is used. The sample is heated to selected temperatures in a furnace with the flow of high-purity argon to avoid oxidation.

### Data availability

The data that support the findings of this study are available from the corresponding authors on request.

## Additional information

**How to cite this article:** Ma, T. *et al*. Highly thermal-stable ferromagnetism by a natural composite. *Nat. Commun.* **8,** 13937 doi: 10.1038/ncomms13937 (2017).

**Publisher's note:** Springer Nature remains neutral with regard to jurisdictional claims in published maps and institutional affiliations.

## Supplementary Material

Supplementary Information Supplementary Figures 1-5 and Supplementary Reference
