# Erratum: Highly thermal-stable ferromagnetism by a natural composite

**DOI:** 10.1038/ncomms14704

**Published:** 2017-03-21

**Authors:** Tianyu Ma, Junming Gou, Shanshan Hu, Xiaolian Liu, Chen Wu, Shuai Ren, Hui Zhao, Andong Xiao, Chengbao Jiang, Xiaobing Ren, Mi Yan

Nature Communications 8: Article number: 13937; DOI: 10.1038/ncomms13937 (2017); Published 01 18 2017; Updated 03 21 2017

The Author Mi Yan was incorrectly omitted from the list of corresponding Authors in the PDF of this Article, and the author Tianyu Ma was incorrectly listed as a corresponding author; the HTML version of the paper was correct from the time of publication. The correct information for correspondence is: ‘Correspondence and requests for materials should be addressed to X.R. ren.xiaobing@nims.go.jp) or to M.Y. mse_yanmi@zju.edu.cn).'

