# Supplementary material for: Highly thermal-stable ferromagnetism by a natural composite
Source: Nat Commun. 2017 Jan 18;8:13937. doi: 10.1038/ncomms13937 (PMC5253650; doi:10.1038/ncomms13937)
Supplement: Supplementary Information — Supplementary Figures 1-5 and Supplementary Reference [file ncomms13937-s1.pdf]

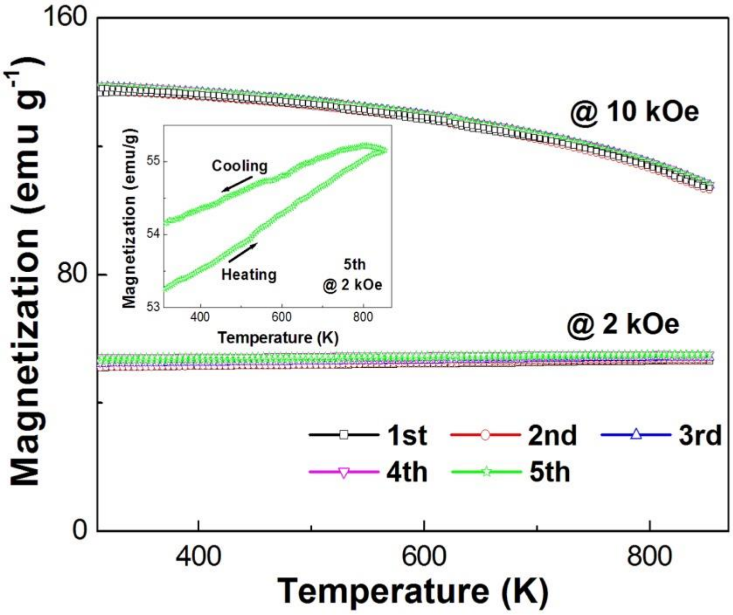

Supplementary Figure 1 | Magnetization stability against thermal cycles. *M*-*T* curves of the $Fe_{73}Ga_{27}$ alloy aged for 12 h at 803 K measured under 2 kOe and 10 kOe for 5 cycles. This sample was not subjected to hot rolling before solution-treating and aging. For a thermal cycle, the sample was heated up to 850 K, then cooled back to room temperature. The inset shows the *M*-*T* curves for the 5th cycle under 2 kOe. It reveals that the magnetization keeps excellent thermal-stability after repeated heating-cooling cycles. Over the temperature range from 310 to 850 K, the magnetizations in both low and high magnetic fields (2 kOe and 10 kOe, respectively) are not deteriorated and are stable up to 5 cycles. Especially, there is no obvious tendency of magnetization deterioration observed for the low magnetic field case upon heating. The magnetization of the sample cooled back to 310 K is a little higher than that of initial state after 4 thermal cycle under magnetic field of 2 kOe (shown in the inset is the 5th cycle), demonstrating the irreversible transformation into the $L1_2$ phase with higher magnetization than that of the BCC phase.

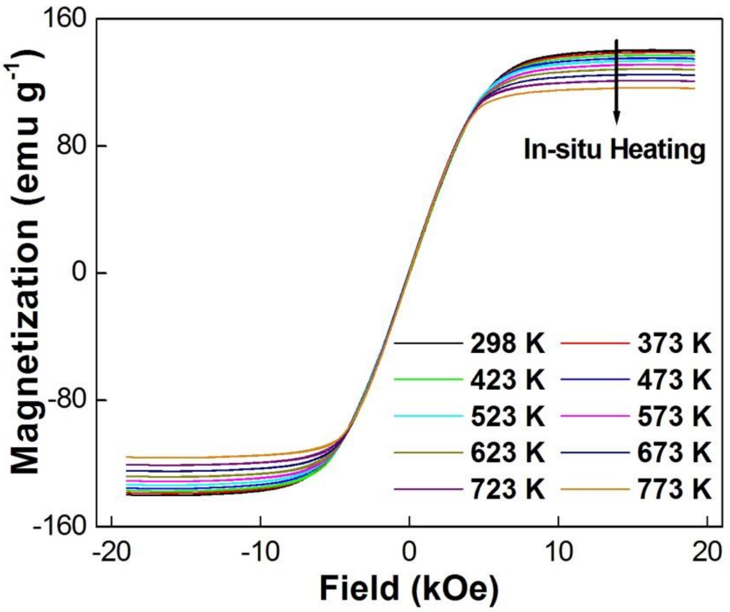

Supplementary Figure 2 | Isothermal magnetizations. *M-H* loops for the $Fe_{73}Ga_{27}$ composite measured upon in-situ heating to different temperatures. It shows that thermally stable magnetization is achieved in such ferromagnetic composite, especially for the low magnetic fields. The magnetization up to 4.5 kOe keeps non-deterioration over the measured temperature range from 298 K to 773 K. Since the magnetostriction of this alloy saturates ~4.5 kOe, the extreme stable magnetization in low magnetic fields then has plenty of potential applications. We should not deny that the magnetization at strong magnetic fields slightly decreases with temperature. The reasons are i) the strong magnetic field could suppress the structural transformation from BCC to $L1_2$ and ii) the untransformed BCC phase and the stable $L1_2$ new phase still obey the Brillouin function.

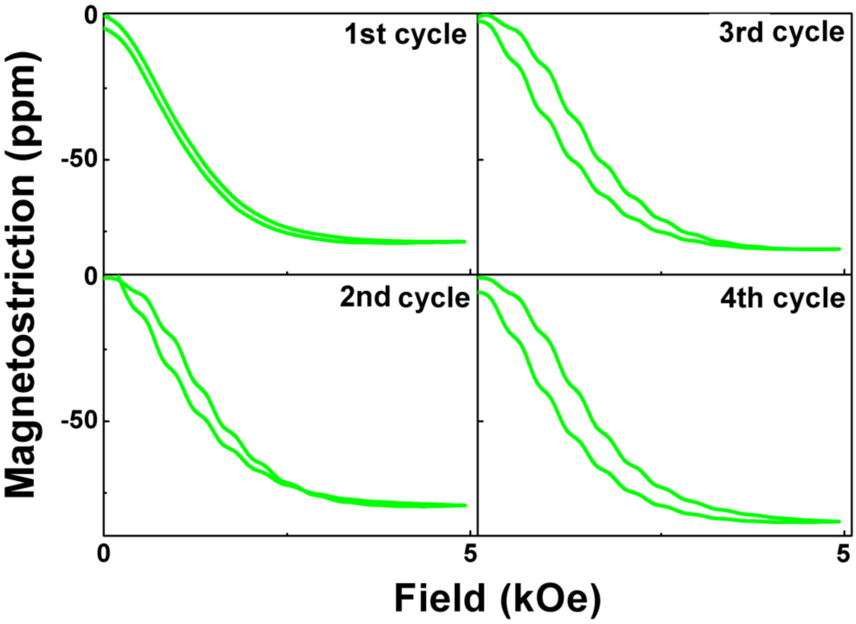

Supplementary Figure 3 | Magnetostriction after thermal cycling. Room temperature magnetostriction vs. magnetic field curves of the $Fe_{73}Ga_{27}$ composite after thermal cycling over the temperature scope from 310 to 850 K, also showing stable magnetostriction performance. As the cycle number increases, the saturation magnetostriction (absolute value) as well as the magnetostriction hysteresis become slightly larger. This is consistent with the enlarged volume fraction of the $L1_2$ phase after thermal cycling. In our recent work,[1] the aging time at 803 K is prolonged to 30 days, the $L1_2$ phase becomes the majority with small amount of the untransformed BCC phase. The magnetocrystalline anisotropy constant and coercivity of the 30-day-aged sample are much larger than the BCC-averaged sample (at the solution-treated state), indicating that the FCC phase also exhibits stronger magnetocrystalline anisotropy constant than the BCC phase. Consequently, the enlarged magnetostriction hysteresis after cycling reflects the strengthened anisotropy of forming more $L1_2$ phase.

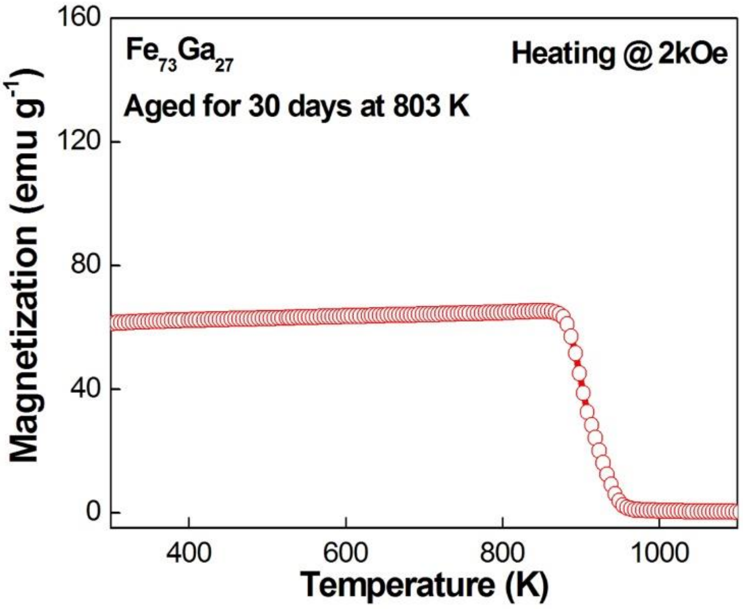

Supplementary Figure 4 | Magnetization stability of long-term aged sample. *M-T* curve of the 30-day-aged $Fe_{73}Ga_{27}$ sample measured under 2 kOe. The sample contains mainly of $L1_2$ phase and a minority of untransformed BCC phase.[1] The two-phase coexistence indicates that complete transformation state is not reached even after aging for 30 days at 803 K. Upon heating, the gradual structural transformation from BCC into FCC still occurs, hence thermally stable magnetization is obtained below Curie temperature, i.e. prior to the $L1_2$ (ferromagnetic) $\rightarrow DO_{19}$ (paramagnetic) transformation. It further demonstrates that the BCC $\rightarrow L1_2$ transformation can compensate for the thermally induced magnetization reduction and implies that the $Fe_{73}Ga_{27}$ alloy aged for 12h at 803 K may have long operating life.

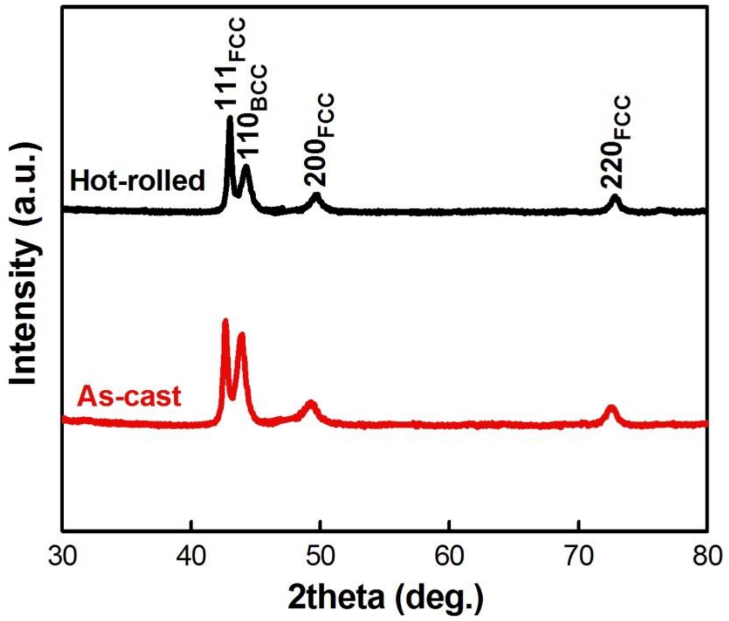

Supplementary Figure 5 | Texture influence. XRD profiles of the hot-rolled (black curve) and the as-cast (red curve) $Fe_{73}Ga_{27}$ samples after solution-treating for 3 h at 1373 K and aging for 12 h at 803 K. The as-cast ingot also bears a bi-phase structure after the same heat-treatments with the hot-rolled sheet. The $\{111\}_{FCC}$ and $\{110\}_{BCC}$ reflections are the strongest ones for BCC and FCC phases in these two samples, respectively. The aged ingot has also similar thermally stable magnetization to the aged sheet. Consequently, the texture has slight influence on the magnetization stability of the Fe-Ga composite. The grain size could be different between the as-cast ingot and the hot-rolled sheet.

Supplementary Reference:

1. Gou, J.M. et al. Tailoring magnetostriction sign of ferromagnetic composite by increasing magnetic field strength. *Appl. Phys. Lett.* **109**, 082404 (2016).
